# Regulation of Small RNAs and Corresponding Targets in Nod Factor-Induced *Phaseolus vulgaris* Root Hair Cells

**DOI:** 10.3390/ijms17060887

**Published:** 2016-06-04

**Authors:** Damien Formey, José Ángel Martín-Rodríguez, Alfonso Leija, Olivia Santana, Carmen Quinto, Luis Cárdenas, Georgina Hernández

**Affiliations:** 1 Centro de Ciencias Genómicas, Universidad Nacional Autónoma de México (UNAM), Avenida Universidad 1001, Cuernavaca 62210, Morelos, Mexico; formey@ccg.unam.mx (D.F.); jamartin@ccg.unam.mx (J.Á.M.-R.); leija@ccg.unam.mx (A.L.); 2 Departamento de Biología Molecular de Plantas, Instituto de Biotecnología (UNAM), Avenida Universidad 2001, Cuernavaca 62210, Morelos, Mexico; olivia@ibt.unam.mx (O.S.); quinto@ibt.unam.mx (C.Q.); luisc@ibt.unam.mx (L.C.)

**Keywords:** common bean, legume-rhizobia interaction, miRNAs, phasiRNAs

## Abstract

A genome-wide analysis identified the set of small RNAs (sRNAs) from the agronomical important legume *Phaseolus vulgaris* (common bean), including novel *P. vulgaris*-specific microRNAs (miRNAs) potentially important for the regulation of the rhizobia-symbiotic process. Generally, novel miRNAs are difficult to identify and study because they are very lowly expressed in a tissue- or cell-specific manner. In this work, we aimed to analyze sRNAs from common bean root hairs (RH), a single-cell model, induced with pure *Rhizobium etli* nodulation factors (NF), a unique type of signal molecule. The sequence analysis of samples from NF-induced and control libraries led to the identity of 132 mature miRNAs, including 63 novel miRNAs and 1984 phasiRNAs. From these, six miRNAs were significantly differentially expressed during NF induction, including one novel miRNA: miR-RH82. A parallel degradome analysis of the same samples revealed 29 targets potentially cleaved by novel miRNAs specifically in NF-induced RH samples; however, these novel miRNAs were not differentially accumulated in this tissue. This study reveals *Phaseolus vulgaris*-specific novel miRNA candidates and their corresponding targets that meet all criteria to be involved in the regulation of the early nodulation events, thus setting the basis for exploring miRNA-mediated improvement of the common bean–rhizobia symbiosis.

## 1. Introduction

MicroRNAs (miRNAs), a class of non-protein-coding RNA (npcRNA) of ~22 nucleotides (nt) in length, play pivotal roles as central regulators of gene expression in plants controlling fundamental processes, such as morphogenesis, development, stress response, and plant-microbe interactions [1]. The plant miRNA precursors (pre-miRNA), generally transcribed by RNA polymerase II, adopt stem-loop structures that are processed by several enzymes including the endonuclease Dicer-like 1 (DCL1) and generate mature miRNAs that are loaded onto a member of the Argonaute (AGO) protein family to assemble the RNA-induced silencing complex (RISC). The miRNA can then play its negative regulatory role by specifically binding a target transcript based on sequence complementarity and, thereby, causing its degradation or its translation inhibition [1,2]. Growing evidence supports the participation of miRNAs in the control of the legume-rhizobia nitrogen-fixing symbiosis [3,4].

A key to success of the large legume family was the evolution of symbiosis with nitrogen-fixing bacteria of the family *Rhizobiaceae* to directly capture atmospheric dinitrogen (N_2_) to support plant growth and productivity. The symbiotic N_2_-fixation occurring in the legume-rhizobia symbiosis takes place in root-developed specialized organs called nodules. Nodulation is a complex process that involves a tight communication between a rhizobia and its specific legume host through molecular signals. The first step in this molecular dialog is the detection by rhizobia of flavonoids secreted from the legume root that triggers the rhizobial biosynthesis of lipochitin-oligosaccharide symbiotic signals known as nodulation factors (NFs). NFs bind to high-affinity receptors and are perceived by the plant mainly in the tip of emerging root hairs (RHs). The presence of compatible rhizobia species and their corresponding NFs are generally sufficient to trigger rhizobial infection and nodule development. The rhizobial infection normally starts by the attachment of the bacteria to the RH tip, giving rise to several plant responses, including changes in actin filaments and vesicle trafficking, as well as formation of pre-infection structures, such as RH deformations (branching, swelling, and curling) that are followed by the development of infection threads. A prerequisite to infection and nodulation processes is the activation of signaling pathways by the NF receptors, which include nuclear Ca^2+^ oscillations, or calcium spiking, and perception/transduction of the calcium spiking signal by different components that, in turn, control the expression of early nodulation genes (for review see [5,6,7,8]).

Studies on miRNAs from different legume species addressed their regulatory role in the nodulation process; however, these are still relatively few (for review see [3,4]). Conserved and legume-specific miRNA families differentially expressed during nodule organogenesis were identified for *Medicago truncatula*, *Lotus japonicus*, soybean (*Glycine max*), and common bean (*Phaseolus vulgaris*) [9,10,11,12,13,14]. However, demonstration of specific functions of legume miRNAs in different stages of the legume-rhizobia symbiosis has been obtained for only few candidates mostly from conserved miRNA families. Regarding studies of legume miRNAs potentially important for the early stages of the symbiosis, Formey *et al*. [13] identified miRNAs from *M. truncatula* roots that respond to treatments of 24 h with purified NFs. In addition, miRNAs differentially expressed in soybean roots inoculated for 3 h with *Bradyrhizobium japonicum* were identified; these include around 120 novel miRNAs [9,12]. More recently, Yan *et al*. [15] analyzed soybean miRNAs that respond to *B. japonicum* inoculation (12 to 48 h) in RHs. This single-cell model with important general roles in water and nutrient uptake, and being the site of rhizobia infection, was successfully used as a single-cell model for systems biology [16]. Transcriptomic, proteomic, and metabolomics analyses from pure preparations of soybean RHs revealed a myriad of soybean molecules, including 114 miRNAs (from these 22 were novel miRNAs) and 405 soybean miRNA targets with potentially relevant roles in the early stages of the rhizobia symbiosis [15,17,18,19,20].

The common bean is the most important legume for human consumption, being the principal source of proteins for millions of people mainly in Latin America and Africa [21]. Current research from our group is focused in the functional characterization of common bean miRNAs as regulators of the legume-rhizobia symbiosis. We demonstrated the pivotal role of the miR172c–AP2-1 node in the regulation of the common bean-*Rhizobium etli* N_2_-fixing symbiosis [22]. This miRNA is a positive regulator of the common bean nodulation. The soybean miR172 has a similar role as a key regulator of the *B. japonicum* symbiosis; the direct transcriptional repression of its AP2 target gene on the *Early Nodulin 40* gene (*ENOD40*) was demonstrated [23,24]. A genome-wide analysis based in the *P. vulgaris* genome sequence, using five small RNA libraries from different plant organs and degradome data, led us to identify the catalog of small RNAs (miRNAs and phasiRNAs) and their associated target transcripts from common bean [14]. Our data revealed newly-identified *P. vulgaris*-specific miRNAs potentially important in the control of the rhizobia-symbiotic process; their functional characterization is one of our main goals. Generally novel family- or species-specific miRNAs have very low expression in a specific tissue or cell type, something that complicates their identification and functional analysis. To this end, in this work we aimed to analyze small RNAs (sRNAs) from common bean RH, a single-cell model, induced with pure *R. etli* NFs, a unique type of signal molecule. Based in soybean research [15,17,19,20,25], we hypothesized that our analysis would allow the identification of novel common bean miRNAs for the regulation of initial stages of the rhizobia symbiosis taking into account that a single cell type rather than an entire tissue avoid potential dilution effects and enables a more sensitive and accurate analysis of plant cell responses to specific stimuli. A high-throughput RNA sequence analysis of sRNA libraries from common bean RH induced for 6 h with pure *R. etli* NF, as compared to control (non-induced) RH, as well as a parallel degradome analysis of the same samples, led to identifying novel miRNAs and their potential targets. This work set the basis for exploring novel common bean miRNA target nodes potentially relevant as regulators of the initial events of the N_2_-fixing symbiosis of this agronomical important legume.

## 2. Results

### 2.1. Common Bean Root Hairs (RHs) Isolation and Response to R. etli Nodulation Factors (NFs)

**Figure 1:**
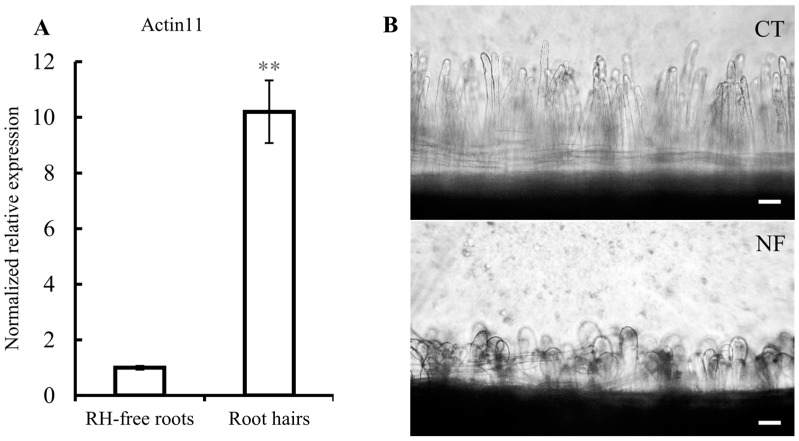

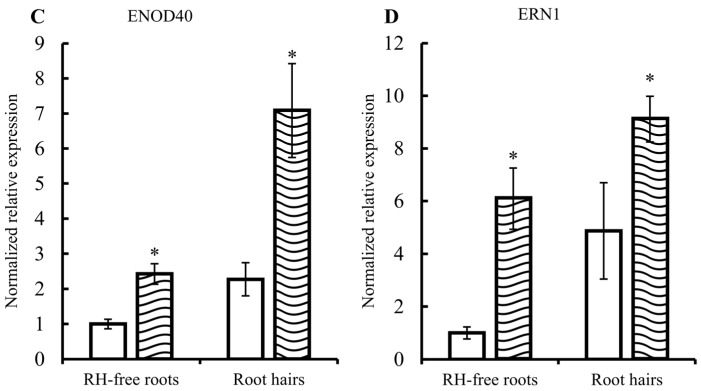
Validation of the common bean root hairs (RH) single-cell system. Relative expression of (**A**) *Actin 11*, (**C**) *ENOD40*, and (**D**) *ERN1*. In **C** and **D**, white and striped histograms represent the mean of expression values from mock-induced and nodulation factors (NF)-induced samples, respectively. Expression values are normalized to the value of the control of RH-free roots that was set to 1. Error bars represent the standard error of the mean of the relative expressions from three biological replicates. Mann-Whitney statistical test: * *p* < 0.05, ** *p* < 0.01; and (**B**) microscopic images showing RH from mock-induced control roots (CT: Control, **top**) and from NF-induced roots (NF: NF-induced, **bottom**). Scale bars: 100 µm.

Soybean RHs were used as a single-cell model for systems biology [16]. In this work we used the single cell-model of common bean RHs, not reported before, to analyze sRNAs that respond to *R. etli* NFs, non-commercial and non-standardized molecules were purified as reported [26]. Thus, an important initial part of this work was the validation of our experimental procedure. To evaluate the adequate high concentration and purity of isolated common bean RHs we quantified the level of *Actin 11* transcripts, known to be specifically abundant in RHs [27]. As shown in Figure 1A *Actin 11* transcript was ten times higher in the RH samples than the samples from roots separated from RHs (hereby denominated as RH-free roots), indicating that the RH single-cells and their corresponding transcripts are significantly more abundant in the RH samples. Our experimental procedure included the induction of common bean roots with pure *R. elti* NF, for 6 h. The microscopic observation of NF-induced *versus* non-induced roots clearly show the expected RH deformation (Figure 1B), thus confirming the effectiveness of the NF treatment used. To assess the expected changes in early-nodulin gene expression triggered by NF perception/signaling, we determined the expression level of the *ENOD40* and *ERN1* (*Ethylene Responsive Factor required for Nodulation 1*) marker genes (Figure 1C,D). These two genes showed higher expression in RH than in RH-free roots and, in addition, a significant increase was observed in both tissues after NF induction. These results validated our method for preparation of concentrated RH samples and their induction by NF.

### 2.2. Sequencing Data

In this work we sequenced small RNA libraries from three biological replicated from NF-induced RH samples (NF1, NF2, and NF3) and control, mock-induced samples (CT1, CT2, and CT3). We obtained an average of 12,672,401 raw reads per library (Table 1). Around 53% of the raw reads were retained after quality cleaning and mapping on the *Phaseolus vulgaris* genome. After the suppression of the redundancy, an average of 1,425,724 unique sequences per library were selected for the small RNA identification processes.

The majority of the sequenced small RNA reads is in a size range of 20–24 nt (Figure S1). As expected with the size distribution of Dicer-processed molecules, the two most abundant size classes of these small RNA are the 21 and 24 nt.

### 2.3. miRNA Identification

Cleaned and mapped sRNA sequences were used to identify previously referenced and novel miRNAs based on the miRDeep-P pipeline [28]. For a better support of identified candidates, ShortStack 3.3 [29] was additionally used in our identification analysis. A total of 207 miRNA precursors were identified, generating 132 unique mature miRNAs (Table 2). Around 33% of the identified precursors were also predicted by ShortStack, something that gives robustness to these candidates. From the 132 mature miRNAs identified in this study, 47 are known miRNAs that are conserved in different plant species (miRBase v. 21, Faculty of Life Sciences, University of Manchester, Manchester, UK), 22 are new isoforms (or family members) of already-referenced miRNAs, 55 are novel miRNAs identified for the first time in this study, and three are new isoforms of novel miRNAs identified previously by Formey *et al.* [14] (Table S1). The sequences of all 63 novel miRNAs were detected in at least three of our analyzed libraries and *ca.* 87% of these were detected in all six libraries. The novel miRNAs are, hereby, denominated as “miR-RHnumber”.

Table 2 shows the comparison of the mature miRNA sequences identified in this work from the single-cell RH system with those we had previously identified from other common bean tissues [14]. The total mature miRNAs previously identified accounted only for 35% of those identified from RH. While a high proportion (79%) of RH-conserved miRNAs were already identified [14], only 8% of the novel miRNAs identified here from RH had been identified in different common bean tissues (Table 2). The latter supports our hypothesis that a single-cell type system avoids signal dilution and enhances the potential to discover new regulatory miRNAs for the early legume-rhizobia interaction.

### 2.4. Prediction and Identification of miRNA Targets

Based on the degradome data from RH and using the psRNATarget [30] prediction tool we were able to predict the targets of the identified miRNAs. An average of 0.6 degradome targets per miRNA were identified (Table 3).

Around one-third of the miRNA have at least one degradome target. The prediction of the targets by computational method based on base pairing with transcripts (psRNAtarget) allowed us to characterize at least one target for 90% of the miRNAs. A mean of 12 targets per miRNA were identified, which is 20 times more than the results obtained by degradome sequencing.

Based on the degradome data, from the 75 identified targets, 56 were identified in the NF-induced samples, 33 in the corresponding controls, and 14 are common between the two libraries (Table S2). Among the 42 specific targets of NF-induced libraries, 29 are cleaved by novel miRNAs, eight are cleaved by new isoforms and five are cleaved by known miRNAs. Compared to the miRNAs identified in five different tissues of *P. vulgaris*, the identification of miRNA targets in RHs reveals a lower proportion of degradome targets per conserved miRNA and a higher proportion for the novel miRNAs, suggesting a higher activity of the novel miRNAs in this particular tissue where the conserved ones are less active.

### 2.5. PhasiRNA Identification

The phasiRNAs are another class of sRNAs with a length of 21, 22, or 24 nt. The ShortStack software allowed identifying a total of 1984 loci, with an average length of 846 nt, potentially-generating phasiRNAs (Table 4 and Table S3). Almost all (98%) of these loci produce 24 nt phasiRNAs, while only 32 and three loci were identified as generating 21 and 22 nt phasiRNAs, respectively.

### 2.6. Reads Differential Expression of Identified miRNAs

**Figure 2:**
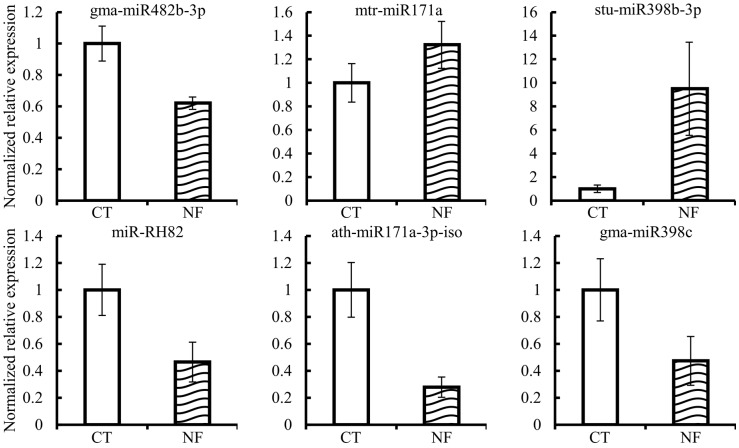
Mature miRNA differentially expressed in NF-induced RH single-cells, based on high throughput sequencing data. White and striped histograms represent the mean of expression values from control (CT, mock-induced) and NF-induced (NF) samples, respectively. Expression values are normalized to the value of the control condition that was set to 1. Error bars represent the standard error of the mean of the three relative expressions from three biological replicates of each treatment. Mann-Whitney statistical test, *p* < 0.05.

We performed statistical analysis of the sRNA-sequences from the three replicates per treatment in order to identify those miRNAs, from the total 132 identified (Table 2), differentially expressed in the NF-induced RHs, as compared to control RHs. Data from Figure 2 show that only six miRNAs presented a significant differential expression (*p* ≤ 0.05) in our conditions, isolated RHs induced by pure NFs (10^−7^ M) over 6 h.

Our analysis identified one novel miRNA, miR-RH82, which showed significant down-regulation upon NF induction. Unfortunately, no potential miR-RH82 targets were identified from our degradome analysis. Bioinformatic analysis led us to predict ten different targets for this novel miRNA, thus, its experimental validation is complicated. From the predicted miR-RH82 targets, three are members of the RNI-like superfamily encoding an F-box protein containing the Leucin Reach Repeats (LRR) domain.

The other five miRNAs differentially expressed in NF-induced RH are members of the miR171, miR398 and miR482 families. Previous knowledge from different legumes indicate the participation of these three miRNA families in the regulation of the nodulation: miR171 regulates the key nodulation transcription factor Nodulation Signaling Pathway 2 (NSP2) [31], miR398 has been measured as down-regulated in *P. vulgaris* root tissues during the first hours of the inoculation by *Rhizobium tropici* [32], and miR482 targets genes involved in plant disease resistance that regulate the establishment of the nodulation [33].

### 2.7. Expression Quantification of Candidate miRNAs and Corresponding Targets

**Figure 3:**
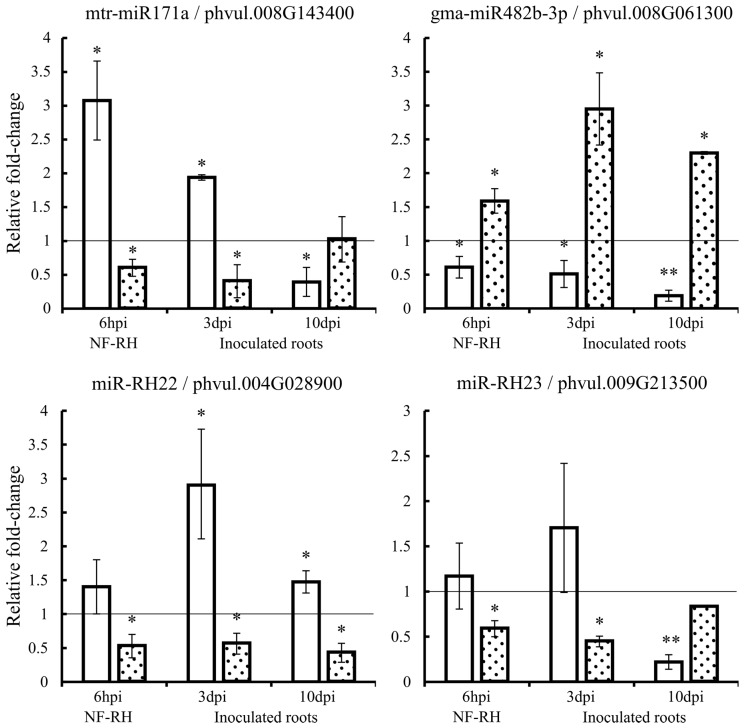
Relative fold-change expression (qRT-PCR) of mature miRNA (white histograms) and the corresponding targets (dotted histograms) in NF-induced RHs at 6 hpi (hours post induction) (NF-RH) and *R. etli*-inoculated roots at 3 and 10 dpi (days post inoculation). Values were normalized to the values of the mock control, set at 1 as indicated by the line. Error bars represent the standard error of the mean of the three relative expressions from three biological replicates. Mann-Whitney statistical test, * *p* < 0.05 & ** *p* < 0.01.

We validated the expression by qRT-PCR of the mtr-miR171a, gma-miR482b-3p, miR-RH22, miR-RH23, and the corresponding predicted targets from the RNA samples used for sequencing (Figure 3). In addition, we determined their expression in *R. etli*-inoculated roots at early stages, three and 10 days post-inoculation (dpi).

For mtr-miR171a, the expression profile was the same at 6 h post-induction (hpi) and 3 dpi with an increased expression of the miRNA and the corresponding decreased expression of the target gene (GRAS family protein). However, at the longer inoculation time point (10 dpi), the expression profiles changed: the decreased expression of mtr-miR171a and the increased expression of the target were observed. For gma-miR482b-3p, the expression profile was similar in NF-induced RHs and in inoculated roots at the two time points analyzed: the miRNA was down-regulated and the corresponding target (Nucleotide Binding Site (NBS)-LRR class protein) was up-regulated. The expression data of miR-RH22 in NF-RH (6 hpi) from Figure 3 validated the sequencing data that showed no differential expression of this novel miRNA but a significant down-regulation of its corresponding target (Toll/interleukin-1 receptor (TIR)-NBS-LRR class protein), according to the degradome analysis. In addition, at 3 and 10 dpi, the miRNA was significantly up-regulated while its target was significantly down-regulated. We observed a similar expression profile for the miR-RH23 with no change of miRNA expression in NF-RH and at 3 dpi but a significant down-regulation of the corresponding target (MEI2-like protein 5). At 10 dpi, the miRNA was significantly down-regulated while the corresponding targets were not significantly down-expressed anymore.

## 3. Discussion

The NFs are molecules secreted by the nitrogen-fixing rhizobia that prepare the plant roots for their colonization by these bacteria [34]. Once perceived, the NFs generate a signaling cascade that induce the expression of various genes including transcription factors such as AP2 or NSP2 [22,31]. These transcription factors are known to be targeted and regulated by miRNA during the establishment of the nodulation [11,22]. To date, many miRNAs have been identified as differentially expressed in nodules compared to roots or during the induction by NFs [9,10,11,12,13,14,15], these include novel miRNAs. In general, novel miRNAs are difficult to identify or analyze because they are known to be expressed at a low level and to be tissue- or cell-specific [14,35]. In this study, we chose to concentrate the signal of tissue-specific miRNAs potentially involved in the early events of the N_2_-fixing symbiosis focusing in one cell type, the RHs, and one type of molecule, the NFs. We sequenced six libraries corresponding to three biological replicates of NF-induced RHs during 6 h and three biological replicates of the mock-induced controls. Previously, Formey *et al.* [13] studied the transcriptional response of miRNAs to the induction of *M. truncatula* roots by NFs. Since these authors did not focus on the RHs, the primary cells to perceive the signal, the majority of the low expressed RH-specific miRNAs could have been diluted and, thus, unidentified. Another group focused on this topic analyzing soybean-isolated RHs but using living bacteria (rhizobia), rather than a signal molecule, for their induction [15]. Thus, to our knowledge, our study on the common bean is the first of the symbiosis field to present small RNAs and their corresponding predicted targets that respond transcriptionally in one specific tissue (RHs) and that are induced by one type of molecule (NFs).

### 3.1. Small RNA Identification

Among the 132 mature miRNAs identified in this study, 69 are known miRNAs or new isoforms of already known miRNA. These are distributed in 33 families: 24 considered as conserved families, from miR156 to miR482, which are present in most angiosperms [36], and nine more particular families, from miR1512 to miR9749. While the miR477 and miR530, so-called highly-conserved miRNAs, were present in the common bean tissues previously studied (Formey *et al.* 2015 [14]) these were absent in the RH single cells; in agreement, these miRNAs were absent in the RHs from soybean [15]. We predicted a member of the tetratricopeptide repeat-like protein and an ABA-inducible BHLH-type transcription factor as targets of miR477 and miR530, respectively [14]. In *Arabidopsis*, these proteins play a crucial role in RH development [37,38], thus their similar role in legume RH is proposed. Our proposition supports the presence of the tetratricopeptide repeat-like protein and an ABA-inducible BHLH-type transcription factor, together with the absence of their negative regulators miR477 and miR530, as observed in common bean and soybean RH. On the other hand, only the miR9749 was exclusively detected in common bean RH cells and not in the other five tissues previously studied [14]. This miRNA targets a member of the pentatricopeptide domain protein family, one of the largest in plants, which is involved in the edition of proteins [39]. It is conceivable that this protein, or another miR9749 target protein not yet identified, have important roles in the RH cells as the corresponding miRNA is specifically expressed in this cell type. Thus, we propose this miRNA as a new candidate for the study of the miRNAs regulation of RH development or function.

### 3.2. New Players in NF Signaling

The sequences we generated from three biological replicates of NF-induced RHs, and the corresponding controls, allowed us to perform statistical analysis of the differential expression of miRNAs. Unexpectedly, we identified only six miRNAs, distributed in four families, which are differentially expressed during NF treatment. The miR171, miR482, and miR398 families include some members involved in the regulation of nodulation; these, as well as their targets, are differentially expressed during the early events of the nodulation in other legumes [11,32,33] something that support our findings in the common bean (Figure 3). Our data revealed only one new miRNA, miR-RH82, that responded to NF induction in RH. This miRNA is common bean-specific and has not been identified in other tissues suggesting it could be RH-specific. The predicted targets of this miRNA are various members of the RNI-like superfamily encoding an F-box protein containing a LRR domain, one of the largest families of protein in plants that are involved in a consequent number of processes, including symbiosis and, particularly, regulation of the nodulation [40,41]. In *L. japonicus*, one member of the F-box protein family (Too Much Love, TML), expressed in root tips and nodules, is involved in the control of the autoregulation of nodulation, regulating the number of nodules [41]. Based on its species specificity, its expression profile and the potential role of its target during the nodulation process, miR-RH82 is a strong candidate to be relevant for the efficiency of the with N_2_-fixing bacteria symbiotic system.

Given the small proportion of miRNAs differentially expressed in our condition, we hypothesize that the action of these is not regulated exclusively at a transcriptional level, but possibly at subsequent steps once the miRNA duplex is produced, *i.e*. the capacity to be methylated by HEN1 [42], and/or the exportation of the duplex to the cytoplasm [43], and/or to be loaded in the protein AGO to form the RISC complex [1]. To investigate this hypothesis, we analyzed the degradome and the predicted miRNA targets derived from these data. As we are mostly interested in the discovery of regulation performed by new *P. vulgaris*-specific miRNAs during NF perception, we selected the targets that are predicted as specifically cleaved by the new NF-induced miRNAs identified in this study. We found 29 target candidates potentially cleaved by 14 newly identified miRNAs. Based on their expression level, their validation by the two miRNA-identification programs and/or the nature of the potential targets, six miRNAs were selected. These candidates did not present a significant response to NF induction but were remarkably expressed in NF-induced RH compared to the other new miRNAs identified. The most highly expressed was the miR-RH22 with an expression mean of 782 reads/libraries. This new miRNA is 22 nt long; it was validated by ShortStack and is quite similar to gma-miR1510-3p, though it did not meet the criteria to be part of this family. In our degradome data, we identified two targets for the miR-RH22, a hypothetical protein and a TIR-NBS-LRR class protein. Members of miR1510 family are known to target transcripts from the NBS-LRR protein family and to generate phasiRNAs from these transcripts [44,45]. The miR1510/NBS-LRR node has been suggested to intervene in the regulation of the early phases of rhizobial colonization in legumes [46,47]. In agreement, in this study we detected, specifically in NF-induced RH, a TIR-NBS-LRR protein transcript cleaved at the miR-RH22 binding site (Table S2) that showed a significant down-regulation in this tissue (Figure 3). We also identified phasiRNAs of 21 nt that may be generated from the same TIR-NBS-LRR protein transcript (Chr04:3128374-3129247, Phvul.004G028900.1, Table S3). This finding leads us to hypothesize that the miR-RH22 is a *P. vulgaris*-specific miRNA, expressed exclusively in RH cells, derived from the miR1510 family, and silencing the production of resistance genes during the NF perception by triggering the generation of 21 nt-phasiRNAs from this same transcript. The phenomenon of decreased expression of defense-related genes in legume roots during early nodulation events has been documented [25,48] and, here, we bring a strong new miRNA candidate that could be one of the fine tuners involved in the regulation of this process in the common bean.

Based in the degradome data, we detected other miRNA/target node candidates. The miR-RH23 is a 21 nt-long new miRNA with an expression mean of 761 reads/library (Table S1). It is a *P. vulgaris*-specific miRNA identified only in the RH tissue by the two miRNA-identification programs. Degradome data obtained for this miRNA revealed that its target transcript, coding for MEI2-like protein 5, was cleaved only in NF-induced RHs (Table S2). This finding is reinforced by the significant down-expression of the corresponding transcript during the first steps of the symbiosis establishment (Figure 3). The MEI2 proteins are RNA binding proteins exerting their regulatory effects through interacting with npcRNAs within ribonucleoprotein particles, and promoting their nuclear or cytoplasmic re-localization. In *Arabidopsis* and yeast, MEI2 interacts with npcRNAs, re-localizes to the nuclei and play pivotal regulatory roles in meiosis [49,50]. In monocots, proteins from the MEI2-like family regulate leaf and flower development [50]. In *M. truncatula*, a member of the MEI2-like protein was downregulated in RHs during *Rhizobia* infection [51]. In addition, the regulation of legume nodulation by npcRNAs interacting with RNA binding proteins has been documented. An example of such regulators is *ENOD40* family that associates with a RNA-binding protein (RBP1), re-localizes to cytoplasmic granules and is an essential regulator for nodule formation [50]. On this basis, we propose that in *P. vulga*ris, a MEI2-like protein expressed specifically in NF-induced RHs, would load an npcRNA to regulate the early steps of nodule formation. Thus, miR-RH23, its target MEI2-like protein, and the possible associated npcRNAs, are new candidates for the identification of key regulators of the early events of symbiotic nitrogen fixation.

In this study, we bring new elements to understand the regulation of the miRNA action other than differential expression, such as downstream events including mature methylation, cytoplasm exportation, and AGO1 differential loading. We also identified small RNAs potentially produced in the common bean RHs during the induction by NFs. Some robust new miRNA candidates and their corresponding targets identified by sRNA and degradome sequencing were highlighted and proposed for their functional characterization to reveal their role in the establishment of the symbiotic N_2_ fixation. These findings permit the development of the depth of knowledge of the mechanisms of the nodulation process regulation and bring new candidates that must be considered for the functional analysis and the improvement of the association between the common bean and rhizobia.

## 4. Experimental Section

### 4.1. Isolation of Nod Factors

The isolation of NFs was conducted as described in Cardenas *et al.* [26]. Briefly, the CE3 pMP604 strain of *Rhizobium etli*, transcriptionally active in a flavonoid-independent manner, was grown in B-medium. Supernatants were separated with 0.2 volume of water-saturated *n*-butanol. The fraction was dried and re-dissolved in 60% aqueous acetonitrile. This mixture was concentrated on an BAKERBOND spe Octadecyl (C18) Disposable Extraction Columns (J. T. Baker, Phillipsburg, NJ, USA) and purified using reversed-phase HPLC, as described in Lopez-Lara *et al.* [52].

### 4.2. Plant Materials

Seeds from the *Phaseolus vulgaris* G19833 genotype were surface sterilized with 1% sodium hypochlorite for 5 min and rinsed with sterile distilled water. Then, the seeds were germinated during three days over humidified paper, at 30 °C in the dark. Germinated seeds (six per tube) were transferred to Falcon^TM^ conical centrifuge tubes that were filled with nitrogen-free Broughton and Dilworth solution [53] and 10^−7^ M NFs or the equivalent concentration of CHAPS (2.6 × 10^−8^%) for the NF-induced or mock-induced (control) treatment, respectively. Seedlings were induced for 6 h at 30 °C in the dark. Three biological replicates of *ca.* 160 plants each were generated. After induction the infection zone of roots was collected in liquid nitrogen and stored at −80 °C. The RHs were separated from the roots by two 15 s vortex treatment as described in Sauviac *et al.* [54].

For three and 10 dpi root samples, germinated seeds were transplanted to individual pots with fresh sterile vermiculite, and immediately each bean plant was inoculated with 1 mL *Rhizobium etli* CE3 saturated culture applied directly to the root. For inoculum preparation, *R. etli* CE3 was grown in Peptone-Yeast extract liquid medium supplemented with CaCl_2_ (7 μM), rifampicin (50 μg/mL), and nalidixic acid (20 μg/mL) at 30 °C to a cell density of 5 × 10^8^ cells per ml. Seedlings in pots were grown in a growth chamber with controlled environment (25–28 °C, 16 h light/8 h dark) and were watered every 2 days with nitrogen-free B and D nutrient solution [53]. For control treatment, non-symbiotic condition, full nutrient B and D solution was used. Roots were harvested at three and 10 dpi.

### 4.3. Library Preparation for High-Throughput Sequencing

Total RNA from RH samples were isolated using a mirVana miRNA isolation kit (Ambion, Foster City, CA, USA) according to the manufacturer’s instructions. RNA integrity and quality were checked on an Agilent Bioanalyzer 2100 (Santa Clara, CA, USA). Total RNA samples were sent to LC Sciences (Houston, TX, USA) for the miRNA library preparation and sequencing. Briefly, sRNA libraries were generated using the Illumina TruSeq Small RNA Preparation Kit (Illunima, San Diego, CA, USA) according to the manufacturer’s instructions. The cDNA libraries were sequenced on an Illumina GAIIx machine (Illunima, San Diego, CA, USA) following the vendor's instruction. Once sequenced, the 3′ adaptor (TGGAATTCTCGGGTGCCAAGG) for sequencing was removed from the raw reads. Then, reads with the length less than 10 and a quality mean lower than 33 were excluded.

### 4.4. Small RNA Identification

miRNAs were identified using the miRDeep-P pipeline, as described in Formey *et al.* [14]. Briefly, precursors were identified with a window size of 250 nt, no mismatch allowed and a number of hits in the genome lower than 40, using the *P. vulgaris* G19833 genome as a reference [55]. The software discarded the precursors overlapping an npcRNA. Only the precursors with a mature miRNA between 18 and 25 nt, wherein the mature miRNA lies within the top 5% of the most expressed sequences in a given library, were selected. Mature miRNAs were compared with *Viridiplantae* miRNAs from miRBase version 21 [56] using the NCBI BLASTn program [57], allowing no mismatches. To identify the miRNAs families and the new isoforms, we used CD-HIT (Cluster Database at High Identity with Tolerance) [58] with at least 84.2% of identity. For the novel miRNAs, only mature sequences for which the best precursor miRDeep score is in the top 5% of the precursor miRDeep scores were kept.

miRNAs and phasiRNAs were identified using ShortStack 3.3 [29] with the default parameters. For miRNAs, only the mature miRNAs between 18 and 25 nt and with less than 40 matches in the genome were selected.

### 4.5. Target Prediction

Two RNA samples, from NF-RH and the corresponding control, were sent to LC Sciences (Houston, TX, USA) for the degradome library preparation and sequencing. Briefly, 5′ RNA oligonucleotide adaptors containing a MmeI site were ligated to RNAs, then reverse-transcribed and amplified by PCR. The library was purified on gel for sequencing on Illumina GAIIx.

Targets were identified as described in Formey *et al.* [14]. Briefly, CleaveLand verion 4 [59] was used to identify the putative target sites from degradome data. The transcripts from the version 1 of the *P. vulgaris* genome [55] were used as target templates. The total set of identified miRNAs ([14], this study) was used as the small RNA candidates and only targets with a *p*-value ≤0.05 were selected. psRNATarget [30] was used to predict putative miRNA targets on the same transcript dataset. Default parameters were used and only the targets with an expectation value lower than three were retained.

### 4.6. Read Differential Expression

Sequences from three biological replicates were used to perform statistical test for the detection of mature miRNAs that present significantly different accumulation in NF-induced RHs *vs.* mock-induced RHs. The number of reads of each miRNA present in each library was normalized according to the formula: (miRNA read number × 1,000,000)/total mapped reads per library. To determine the significance difference, we applied a Mann-Whitney test and only selected those with *p*-value ≤0.05, when comparing the two samples.

### 4.7. Expression Analyses

Total RNA extractions of *R. etli* inoculated roots were performed as described for the library preparation. One µg of total RNA from each sample obtained from three or 10 dpi roots was polyadenylated and reversely-transcribed for use in two-step qRT-PCR using the NCode miRNA First-Strand Synthesis and qRT-PCR Kits (Invitrogen, Carlsbad, CA, USA) according to the manufacturer's instructions. The sequences of oligonucleotide primers used are provided in Table S4. Reactions were analyzed in a real-time thermocycler Applied Biosystem 7300 (Foster City, CA, USA). Two technical replicates were performed for each reaction. Relative expression was calculated with the “comparative Ct method” and normalized with the geometrical mean of three housekeeping genes (*HSP*, *MDH*, and *UBQ9*) [60], for the mRNA transcripts, and the U6 sRNA, for the miRNAs. A Mann-Whitney statistical test was performed to evaluate the significantly different expression mean from three biological replicates from inoculated *vs.* mock-inoculated roots.

## Figures and Tables

**Table 1:** Statistics of the small RNA libraries.

Libraries	Raw Reads	Cleaned Reads	Quality Filtered Reads	Mapped Reads	Non-Redundant Reads
*CT1*	11,726,478	9,264,227	9,224,743	6,319,332	1,584,299
*CT2*	12,774,551	8,809,808	8,772,738	5,689,822	1,426,947
*CT3*	13,149,010	11,308,995	11,261,076	6,661,621	1,159,340
*NF1*	15,495,403	11,409,546	11,360,872	7,745,661	1,485,625
*NF2*	12,603,665	10,968,015	10,921,075	7,476,758	1,474,937
*NF3*	10,285,303	9,026,291	8,987,972	6,122,331	1,423,194

**Table 2:** Statistics of the identified precursors and their corresponding mature miRNAs.

miRNA Class	Precursors of miRNAs	Mature miRNA	Mature miRNA Previously Identified by Formey *et al.* 2015 [14]	Identification Validation by ShortStack (%)
Conserved	80	47	37	29 (62)
New isoforms	37	22	4	6 (27)
Novel	87	60	5	7 (12)
New isoforms of novel	3	3	-	2 (67)
Total	207	132	46	44 (33)

**Table 3:** Overview of the miRNA target prediction.

miRNA Class	Targets Predicted by psRNAtarget (Previously Identified by Formey *et al.* 2015 [14])	% of miRNAs with Predicted psRNAtarget Target	Targets Predicted by Degradome Data (Previously Identified by Formey *et al.* 2015 [14])	% of miRNAs with Target Predicted by Degradome Data
Conserved	11 (8.8)	97.5	0.4 (1.9)	32
New isoforms	9	86	0.3	60
Novel	13 (5.8)	93	0.7 (0.3)	37
New novel isoforms	6	100	0	0
Total	12 (6.5)	90	0.6 (1)	33

**Table 4:** Number and size of identified phasiRNAs according to their size.

Dicer Call (nt)	Number of PhasiRNAs	Phase Size (bp)
*21*	32	730
*22*	3	1170
*24*	1949	638

## Supplementary Materials

Supplementary materials can be found at http://www.mdpi.com/1422-0067/17/6/887/s1.
