# Supplementary material for: Regulation of Small RNAs and Corresponding Targets in Nod Factor-Induced Phaseolus vulgaris Root Hair Cells
Source: Int J Mol Sci. 2016 Jun 4;17(6):887. doi: 10.3390/ijms17060887 (PMC4926421; doi:10.3390/ijms17060887)
Supplement: Supplementary file 1 [file ijms-17-00887-s001.zip › ijms-131623-Supplementary Material/ijms-131623-supplementary figure.pdf]

Supplementary Materials: Regulation of Small RNAs and Corresponding Targets in Nod Factor-Induced *Phaseolus vulgaris* Root Hair Cells

Damien Formey, José Ángel Martín-Rodríguez, Alfonso Leija, Olivia Santana, Carmen Quinto, Luis Cárdenas and Georgina Hernández

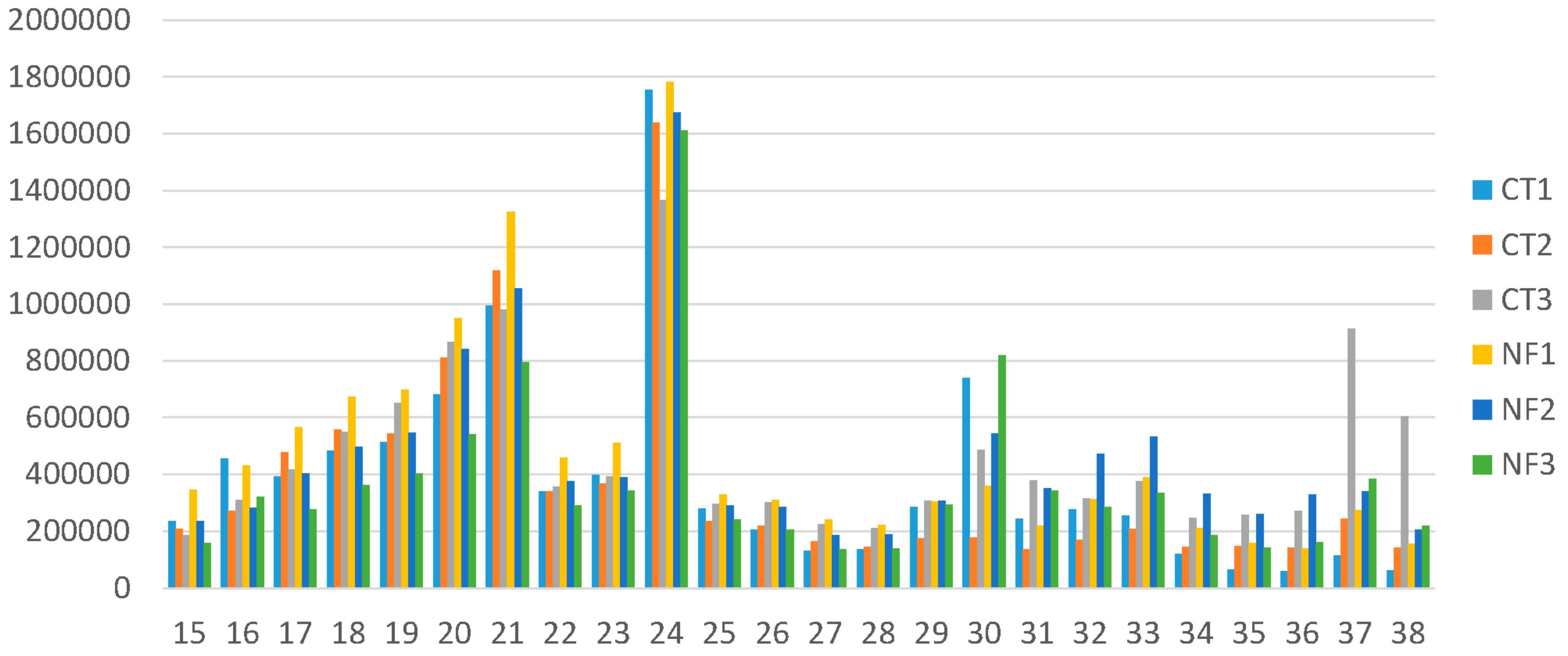

Figure S1. Size distribution of the sequencing reads of each library. *X* axis represents the read sizes (nt) and the *Y* axis represents the number of reads for each read size. The 6 histograms represent the 6 libraries sequences.
